# Erfassung und Operationalisierung des Merkmals „Geschlecht“ in repräsentativen Bevölkerungsstichproben: Herausforderungen und Implikationen am Beispiel der GeSiD-Studie

**DOI:** 10.1007/s00103-021-03440-8

**Published:** 2021-10-15

**Authors:** Carolin Muschalik, Mirja Otten, Johannes Breuer, Ursula von Rüden

**Affiliations:** 1 Referat „Evaluation, Methoden, Forschungsdaten“, Bundeszentrale für gesundheitliche Aufklärung (BZgA), Maarweg 149–165, 50825 Köln, Deutschland; 2 Referat „Sexuelle Gesundheit, Prävention von HIV und anderen STI“, Bundeszentrale für gesundheitliche Aufklärung (BZgA), Maarweg 149–165, 50825 Köln, Deutschland

**Keywords:** Geschlecht, Gender, Geschlechtsidentität, Repräsentativbefragungen, Operationalisierung, Sex, Gender, Gender identity, Representative surveys, Operationalization

## Abstract

Repräsentative quantitative Surveys erheben das Geschlecht der Teilnehmenden, um geschlechtsspezifische Analysen im Hinblick auf die jeweiligen Fragestellungen zuzulassen und Rückschlüsse auf die Populationen zu ziehen. Dies ist wichtig, um zielgruppenspezifische Informationen und Angebote zu entwickeln. Doch obwohl Geschlecht nicht mehr ausschließlich als ein binäres Konstrukt betrachtet wird, wird es noch oft durch eine binäre Variable mit den Antwortmöglichkeiten Frau/Mann oder weiblich/männlich erhoben. In diesem Artikel erörtern wir, warum dieses Vorgehen veraltet bzw. unvollständig und eine Abkehr von diesem Ansatz wichtig und notwendig ist. Anhand der GeSiD-Studie zu „Gesundheit und Sexualität in Deutschland“ zeigen wir in diesem Diskussionsbeitrag exemplarisch auf, wie Geschlecht anhand eines zweistufigen Modells erhoben werden kann, bei dem im ersten Schritt das bei der Geburt zugewiesene Geschlecht und im zweiten Schritt die subjektive Geschlechtszugehörigkeit abgefragt wird. Gleichzeitig erörtern wir die Herausforderungen, die dieser Ansatz mit sich bringt. Die Erfahrungen aus der GeSiD-Studie setzen wir in einen größeren Kontext und diskutieren die Implikationen und Möglichkeiten zur Operationalisierung von Geschlecht in repräsentativen Befragungen.

## Abstract (en)

Representative quantitative surveys assess the participants’ gender in order to allow for gender specific analyses of the research questions and to draw conclusions about the populations. This is important in order to develop information and interventions tailored to the target groups. However, although gender is no longer considered exclusively as a binary construct, it is still often surveyed through a binary variable with the response options woman/man or female/male. In this article, we discuss why this approach is outdated or incomplete and why a move away from this approach is important and necessary. Using the GeSiD study on “Health and Sexuality in Germany” as an example, in this discussion post we demonstrate how gender can be assessed by means of a two-step model in which the first step assesses gender assigned at birth and the second step the subjective gender affiliation. At the same time, we discuss the challenges that this approach brings along. We place the results of the GeSiD study into a larger context and discuss the implications and possibilities for operationalizing gender in representative surveys.

## Einleitung

Bevölkerungsweite Repräsentativerhebungen sind ein wichtiges Instrument empirischer Sozialforschung. Sie werden weltweit zur Bearbeitung von Fragestellungen in unterschiedlichen Gesellschafts- und Lebensbereichen genutzt, z. B. im Kontext von Arbeitsmarktsituationen (z. B. [1, 2]), Bildungschancen (z. B. [3, 4]) oder Religion (z. B. [5]). Auch im Gesundheitsbereich sind epidemiologische Studien sowie Surveys zur Gesundheitsberichterstattung und zum Gesundheits- und Sexualverhalten eine wichtige Informationsquelle. Aktuelle Beispiele aus dem deutschsprachigen Raum sind die Gesundheitsberichterstattung des Robert Koch-Instituts (RKI) sowie repräsentative Surveys zu Themenschwerpunkten körperlicher und psychischer Gesundheit von Kindern, Jugendlichen und Erwachsenen [6], Wiederholungsbefragungen der Bundeszentrale für gesundheitliche Aufklärung (BZgA) etwa zu den Themenkomplexen Rauchen, Alkoholkonsum und Drogenaffinität [7], zum Infektionsschutz [8], zur Organ- und Gewebespende [9], zu HIV und anderen sexuell übertragbaren Infektionen [10] sowie Erhebungen der Bundesanstalt für Arbeitsschutz und Arbeitsmedizin (BAuA) zu Zusammenhängen von Arbeit und mentaler Gesundheit (z. B. [11]). Ergebnisse solcher Untersuchungen können Aufschluss geben zu Prävalenzen von Gesundheitsproblemen und Krankheitsbildern, Gesundheitsverhalten, gesundheitsassoziierten Risikofaktoren, Gesundheitschancen und gesundheitsbezogener Versorgung. Diese Aufschlüsse sind notwendig, um gezielte Maßnahmen für vorherrschende Gesundheitsprobleme angepasst an die spezifischen Zielgruppen gestalten und bereitstellen zu können. Es hat sich gezeigt, dass dieser zielgruppengerechte Ansatz (eng.: „tailored approach“) notwendig ist, um gewünschte positive Veränderungen, beispielsweise im Gesundheitsverhalten, herbeizuführen [12]. Gesundheitsforschung *ohne* Berücksichtigung dieser Diversität bei der Erhebung, Auswertung und Interpretation von Gesundheitsdaten würde eine verzerrte Informationsgrundlage liefern. Auf dieser Basis wäre die Konzeption zielgruppengerechter und nachhaltiger Präventions- und Versorgungsangebote nicht nur deutlich erschwert, sondern auch der Erfolg dieser Angebote gefährdet, da diese nicht auf die Einzigartigkeit und jeweiligen Bedürfnisse der Zielgruppen abgestimmt wären [12].

Ein zentrales Differenzierungskriterium sowohl bei der Erhebung als auch bei der Auswertung gesundheitsbezogener Surveys ist das Geschlecht. Häufig gibt es auch geschlechtsspezifisch unterschiedliche Fragesets oder Fragebögen, eben weil Menschen aufgrund körperlicher Unterschiede unterschiedliche Krankheiten entwickeln [13, 14], unterschiedliche Symptomatiken für dieselben Krankheitsbilder berichten [15] oder sich im gesundheitsbezogenen Verhalten deutlich unterscheiden. Regelmäßig wird das Merkmal Geschlecht dabei binär als weiblich/männlich oder Mann/Frau erhoben. Dies impliziert eine biologische Definition von Geschlecht und zielt auf eine Unterscheidung nach biologischen Geschlechtsmerkmalen ab. Dieses Geburtsgeschlecht oder juristische Geschlecht wurde bis 2018 amtlich in der Geburtsurkunde und im Personalausweis dokumentiert und spiegelt sich zumeist in einem geschlechtsspezifischen Vornamen wider. Durch eine Abfrage des Merkmals Geschlecht, die als Antwortmöglichkeiten ausschließlich „weiblich“ und „männlich“ vorsieht, reproduziert sich jedoch ein statisches Modell der Zweigeschlechtlichkeit, welches als veraltet oder zumindest als unvollständig kritisiert werden muss: Geschlecht ist nicht länger statisch nur als binär zu klassifizieren. Inter*/Intersexualität und Intergeschlechtlichkeit bezeichnen etwa Varianten der Geschlechtsentwicklung auf der Ebene des biologischen Körpers, während mit Trans*/Transsexualität und Transgender, aber etwa auch mit Genderfluidität (immer wieder ein anderes Geschlecht) und agender (ungeschlechtlich/geschlechtslos) auf der Ebene der Geschlechtsidentität Varianten präsent sind, die über ein statisches und binäres Geschlechtermodell hinausgehen. Diese Vielfalt und ihre politische sowie juristische, aber auch medizinische Anerkennung sind äußerst wichtig.

Dass das Bewusstsein für die Geschlechtervielfalt in der westlichen Welt in den letzten Jahren gestiegen ist, zeigt sich u. a. daran, dass Länder wie Dänemark, Niederlande, Kanada oder Australien es ihren Bürger:innen seit einigen Jahren ermöglichen, neben den Kategorien „weiblich“ oder „männlich“ ein „X“ als Geschlechterkennzeichnung in ihrem Pass zu wählen. Auch in Deutschland gibt es seit dem 01.11.2013 mit der Änderung des Personenstandsgesetzes diese Option. Seit dem Jahr 2018, mit dem Gesetz zur Änderung der in das Geburtenregister einzutragenden Angaben, gibt es eine erweiterte Option, Geschlecht im eigenen Ausweis kenntlich zu machen, nämlich den Eintrag „männlich“, „weiblich“, „divers“ oder „kein Eintrag“. Spätestens seit dieser Novelle des Personenstandsgesetzes und der Einführung des dritten Geschlechtseintrags „divers“, muss sich auch die Survey-Forschung in Deutschland die Frage stellen, wie Geschlecht in Surveys am besten operationalisiert und wie es analysiert und repräsentiert werden kann.

Zunächst lässt sich feststellen, dass die Problematik in der empirischen Forschung noch weitgehend ignoriert wird. So verdeutlichte ein Review über 106 veröffentlichte psychologische Studien aus den Jahren 2016–2018, dass in 76 % der durchgeführten Studien das Geschlecht der Teilnehmenden weiterhin als binäres Konstrukt (weiblich/männlich) erhoben wurde [16]. Diese Praxis folgt zum einen nicht den ethischen Prinzipien der Wissenschaft, der Integrität und des Respekts [16]; zum anderen kann für nichtbinäre Personen die binäre Geschlechtsfrage als Identitätsverleugnung empfunden werden [17]. Die daraus resultierenden, wenn auch unbeabsichtigten Verletzungen und Diskriminierungen, zu denen auch „Misgendering“ (der falsche Gebrauch von Pronomen oder anderen geschlechtsspezifischen Wörtern) und „Trans-erasure“ (die Verneinung von transgeschlechtlichen Personen) gehören, sind gut dokumentiert [18].

Der Zusatz einer dritten Geschlechtskategorie („divers“) in Befragungen mag zunächst als ein einfacher und pragmatischer Ansatz erscheinen. Jedoch stößt der Zusatz einer dritten Kategorie „divers“ auf neue vielfältige Probleme. „Divers“ ist zwar als juristische Kategorie eingeführt, umfasst aber verschiedenste Varianten der Geschlechtsentwicklung und wird der Geschlechtervielfalt nicht gerecht. Handelt es sich bei nichtbinären Personen um inter*/intersexuelle Personen, die einen dritten, juristischen Geschlechtseintrag haben, oder um trans*/transsexuelle Personen, die sich vor, nach oder in der Transition befinden? Darüber hinaus würden Personen, die sich als genderqueer (weder ganz/immer weiblich, noch ganz/immer männlich), genderfluid, bigender (doppelgeschlechtlich), trigender (sich mit 3 Geschlechtern identifizierend) und agender identifizieren, in eine Art „Sammelbecken“ fallen, aus dem man keine Rückschlüsse darüber ziehen kann, ob und, wenn Ja, welche Unterschiede es zwischen den verschiedenen diversen Gruppen hinsichtlich spezifischer Fragestellungen gibt. Dies ist allerdings wichtig und notwendig, um zielgruppenspezifische Informationen und Maßnahmen entwickeln und bereitstellen zu können.

Einige wenige Studien haben bereits angedeutet, dass es sich bei der Gruppe „divers“ keineswegs um eine homogene Gruppe handelt, sondern dass diese heterogen ist und Subgruppen sich in bestimmten Merkmalen deutlich voneinander unterscheiden, wie zum Beispiel in der subjektiven Lebensqualität [19], dem Ausüben riskanter Verhaltensweisen [20], in Gesundheitsproblemen und in ihrem Bedürfnis nach adäquater, diskriminierungsfreier medizinischer Gesundheitsversorgung [21].

Ohne weitere Antwortoption und wenn die befragte Person die Frage nicht überspringen kann, ist davon auszugehen, dass die genannte Personengruppe gezwungenermaßen eine der beiden binären Antwortoptionen auswählt, was zu methodologischen Problemen und zu geschlechtsspezifischen Fehlklassifizierungen führen kann [22]. Auch ein Abbruch der Befragung ist als Konsequenz denkbar. Selbst wenn trans* Personen eine binäre Geschlechtsabfrage eindeutig beantworten können, würden diese sich aufgrund ihrer Angaben nicht als transgeschlechtlich identifizieren lassen. Aufgrund dieser Schwierigkeiten fordert die American Psychological Association explizit dazu auf, in der Wissenschaft: (a) Geschlecht als nichtbinäre Kategorie anzuerkennen und (b) die binäre Geschlechtserfassung zugunsten von genaueren und inklusiven Messungen zu verwerfen [23].

Es stellt sich die Frage, wie das Vorhaben einer inklusiven Erfassung von Geschlecht wissenschaftlich umgesetzt werden kann: Wie können empirische Studien oder spezifisch repräsentative Surveys so gestaltet werden, dass dem ethischen Aspekt der Geschlechtervielfalt methodisch Rechnung getragen wird? Welche Herausforderungen ergeben sich in diesem Zusammenhang?

Am Beispiel der GeSiD-Studie zu „Gesundheit und Sexualität in Deutschland“, der ersten umfassenden Erhebung zur Erwachsenensexualität in Deutschland aus dem Jahr 2019, wollen wir aufzeigen, welche Facetten von Geschlecht in Surveys erhoben werden könnten, welche Herausforderungen sich bei der Auswertung und Interpretation der Daten stellen und welche Implikationen sich dadurch für die Entwicklung von Repräsentativerhebungen ergeben.

## Erfassung des Merkmals „Geschlecht“ in der GeSiD-Studie

Im Rahmen der GeSiD-Studie zu „Gesundheit und Sexualität in Deutschland“ wurden zwischen Oktober 2018 und September 2019 mittels persönlicher Interviews 4955 Erwachsene im Alter von 18–75 Jahren befragt. Genutzt wurde eine Einwohnermeldeamtsstichprobe, die in einem zweistufigen, geschichteten Zufallsverfahren ausgewählt wurde. In der Stichprobe ist die Gruppe der jungen Erwachsenen (18- bis 35-Jährige) überrepräsentiert (Oversampling), weil ihr Gesundheits- und Sexualverhalten als besonders relevant eingeschätzt wurde.

Der Fragebogen umfasst 263 Fragen in 14 Themenbereichen. Obwohl die hier diskutierten Implikationen der Gendervielfalt den Studiendurchführenden bewusst waren, wurden Fragebogenversionen für Männer und Frauen eingesetzt. Dies hängt nicht zuletzt mit der Form der Gewinnung der Stichprobe über Einwohnermeldeämter mit binärer Geschlechterzuordnung zusammen. Die Interviews wurden laptopgestützt in den Wohnräumen der Befragten durchgeführt, wobei ein großer Teil der Befragung als Selbstausfüllteil angelegt war, und dauerten durchschnittlich etwa 50 min. Die Interviews wurden von geschulten Interviewer:innen des sozialwissenschaftlichen Instituts KANTAR durchgeführt, wobei aufgrund des sensiblen Themas „Sexualität“ die Frauen von Frauen und die Männer von Männern befragt wurden. Auch hier ergab sich durch die binäre Zuordnung bei der Gewinnung der Teilnehmenden keine Verfahrensweise, die gendersensibler wäre. Für die Durchführung der Studie liegt ein positives Ethikvotum der Hamburger Psychotherapeutenkammer zum GeSiD-Studienprotokoll vor (Referenznummer 07/2018-PTK-HH). Eine vollständige Beschreibung von Studiendesign, Erhebungsmethodik, Teilnahmequote und Stichprobenzusammensetzung findet sich bei Matthiesen et al. [24].

Die Erfassung des Merkmals „Geschlecht“ erfolgte in mehreren Schritten. Zunächst wurde mithilfe von Namen und Anschrift der Befragten das Geschlecht aus den Daten der Einwohnermeldeämter abgefragt. Diese von den Einwohnermeldeämtern übermittelte Geschlechterangabe war wichtig, um (1) das Geschlecht der interviewenden Person zu bestimmen, weil Frauen von Frauen und Männer von Männern interviewt werden sollten, und (2) steuerte bei der Datenerhebung die Auswahl der Fragebogenversion (Form für Männer oder Form für Frauen) sowie die entsprechende Filterführung der computergestützten Eingabemaske. Keines der Einwohnermeldeämter übermittelte den Geschlechtereintrag „divers“, was daran liegt, dass das entsprechende Gesetz erst am 01.01.2019 in Kraft trat und unsere Bruttostichprobe zu diesem Zeitpunkt bereits gezogen war. Befragte, deren gelebtes Alltagsgeschlecht nicht der übermittelten Angabe entspricht, hatten die Möglichkeit, zu Beginn des Interviews die Geschlechterangabe (und damit den verwendeten Fragebogen) in Absprache mit dem/der Interviewer:in zu verändern – hiervon machte allerdings niemand Gebrauch.

**Figure 1:**
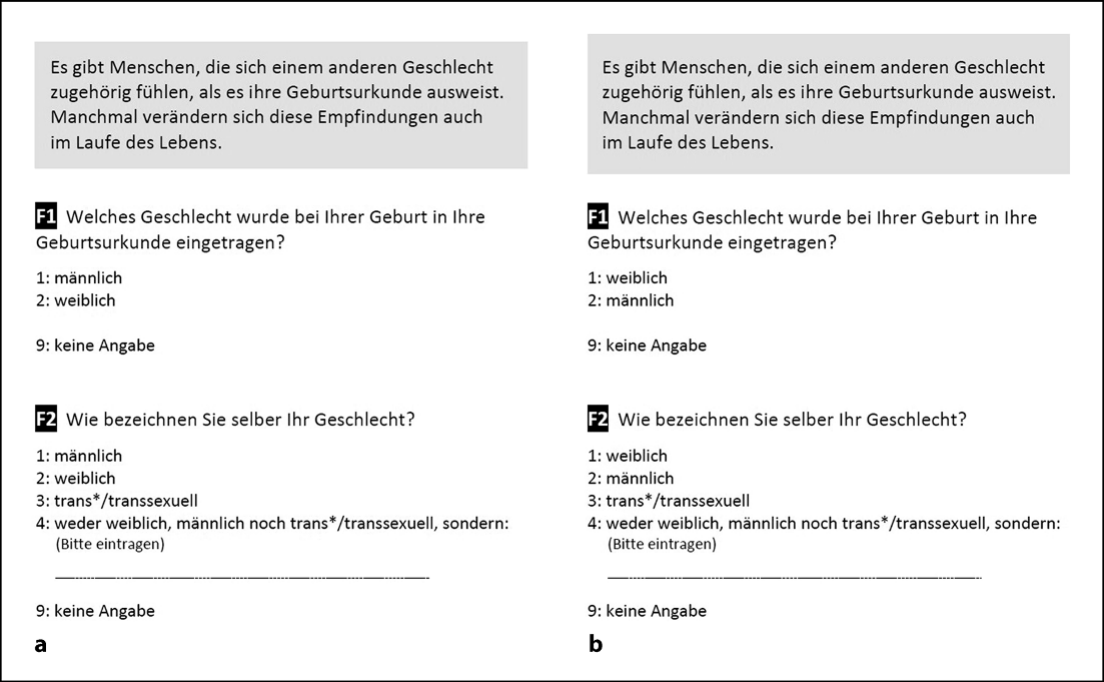

Im Verlauf der Befragung wurden – basierend auf der Empfehlung der GenIUSS Group [25] – alle Teilnehmenden nach dem bei der Geburt zugewiesenen Geschlecht und nach der subjektiven Geschlechtszugehörigkeit zum Befragungszeitpunkt gefragt. Abb. 1 zeigt den Wortlaut der Abfrage des Geburtsgeschlechts (F1) und der Geschlechtszugehörigkeit (F2).

Für den vorliegenden Beitrag wurden Variablen zum juristischen, biologischen und selbstbezeichneten Geschlecht ausgewertet. Für diese Variablen wurden mit der Software IBM SPSS Statistics 26.0 (Modul „Complex Samples“) deskriptive Kennwerte der oben genannten Variablen berechnet.

## Auswertung der Angaben zu „Geschlecht“ in der GeSiD-Studie

### Juristisches Geschlecht.

Von den 4955 Befragten wurden 47,1 % (*n* = 2336) als Männer und 52,9 % (*n* = 2619) als Frauen durch das Einwohnermeldeamt geführt. Für keine der befragten Personen wurden diese Daten vom Interviewer oder durch den Befragten selbst korrigiert. Nicht berücksichtigt wurde die Geschlechtskategorie „divers“, weil deren Einführung erst nach Beginn der Erhebung realisiert wurde.

### Biologisches Geschlecht.

99,4 % der Befragten (*n* = 4926) gaben als ihre Geschlechtszugehörigkeit zum Befragungszeitpunkt das Geschlecht an, das auch bei der Geburt in ihrer Geburtsurkunde angegeben war (juristisches Geschlecht). Ein vom juristischen abweichendes Geschlecht zum Befragungszeitpunkt gaben 0,3 % der Befragten an (*n* = 15). 14 Befragte haben zu dieser Frage keine Angabe gemacht (0,3 %).

### Selbstbezeichnetes Geschlecht.

99,4 % der Befragten (*n* = 4925) bezeichneten ihr Geschlecht als männlich/weiblich kongruent zu ihrem biologischen Geschlecht. 0,1 % der Befragten ordneten sich zwar den binären Kategorien weiblich/männlich zu, sind bei der Angabe aber inkongruent zu ihrem biologischen Geschlecht (*n* = 7). 2 Personen (< 0,1 %) bezeichneten sich als transsexuell, weitere 4 Personen (0,1 %) als keiner vorgenannten Kategorien zugehörig, sondern als (1) „genderfluid“, (2) „Mensch in weiblichem Körper“, (3) „Individuum“ oder (4) „… äußerlich … als Frau … Interessen und Aktivitäten ähneln eher denen der Männer“. Auch hier hat ein geringer Teil der Befragten keine Angabe gemacht (0,3 %; *n* = 17).

Zusammenfassend können wir festhalten: Bei 99,05 % der Befragten sind die Angaben zum Geburtsgeschlecht und zum selbstbezeichneten Geschlecht mit den Angaben vom Einwohnermeldeamt deckungsgleich. Bei insgesamt 47 Befragten kam es jedoch zu verschiedenen Abweichungen (Tab. 1).

**Table 1:** 

Geschlecht lt. EWMA (u. Fragebogen)	Geschlecht lt. Geburtsurkunde	Wie bezeichnen Sie selber Ihr Geschlecht?				
		Männlich	Weiblich	Trans*/transsexuell	Sonstiges	Keine Angabe
Männlich	Männlich	2312	3	2	0	2
	Weiblich	4	1	0	0	2
	Keine Angabe	3	0	0	0	5
Weiblich	Männlich	0	7	0	0	1
	Weiblich	3	2593	0	4	4
	Keine Angabe	0	3	0	0	3

*EWMA* Einwohnermeldeamt

Eine eingehende Einzelfallanalyse dieser 47 Befragten zeigt, dass nur ein sehr kleiner Teil klar als trans*/transsexuell zu identifizieren ist, ein größerer Teil bleibt unklar und könnte auf Eingabefehler zurückzuführen sein (teils aufgrund von Sprachbarrieren).

Für einen Großteil der Analysen der GeSiD-Daten wurde somit wiederum die dichotome Variable „Geschlecht“ verwendet. Die Variable entspricht den von den Einwohnermeldeämtern übermittelten Geschlechterangaben. Da für viele der Fragestellungen die binäre Zuordnung als gesellschaftliche Strukturkategorie in Bezug auf Sexualität eine hohe Erklärungskraft besitzt, konnten in der Studie aufschlussreiche Ergebnisse ermittelt werden. Gleichwohl erlaubt das Studiendesign keine Auswertungen die der postulierten Gendervielfalt gerecht würden.

## Implikationen für die Forschungspraxis

Nur ein sehr kleiner Anteil der Befragten in GeSiD ordnet sich nicht der weiblichen und männlichen Kategorie zu. Dies bedeutet, dass eine differenzierte Darstellung weiterer Geschlechtergruppen hinsichtlich der Variablen zum Gesundheits- und Sexualverhalten aus methodischen Gründen keine Rückschlüsse auf die Grundgesamtheit dieser Gruppen ermöglicht und dass der Vergleich mit anderen Befragtengruppen nicht möglich ist. So haben sich nur 2 der insgesamt fast 5000 befragten Personen als trans*/transsexuell identifiziert. Selbst wenn die Personen, bei denen unterschiedliche Angaben bzgl. Geburtsgeschlecht und eigener Geschlechtsbezeichnung vorliegen, dieser Kategorie zugeordnet würden (was nicht legitim wäre), könnten keine statistisch begründeten Vergleiche zwischen trans*/transsexuellen Menschen und anderen Bevölkerungsgruppen in Bezug auf das Gesundheits- und Sexualverhalten angestellt werden. Eine weitergehende Untersuchung dieser beiden Datensätze würde einer Einzelfallstudie gleichkommen, die von der Einverständniserklärung der Surveys nicht abgedeckt ist, da regelmäßig eine aggregierte Datenauswertung zugesichert wird, die Rückschlüsse auf einzelne Personen verhindert.

Das stellt die Survey-Forschung vor ein Dilemma: Einerseits ist die differenzierte Erhebung des Merkmals „Geschlecht“ aus ethischen und juristischen Gründen sowie zur Beantwortung von Forschungsfragen von großer Bedeutung. Andererseits erscheint die Verwendbarkeit der erhobenen Daten fraglich, da Informationen und Unterschiede zwischen allen Gruppen nicht vollständig abgebildet werden können. Vor diesem Hintergrund lassen sich für die Forschungspraxis die folgenden 6 Implikationen zusammenfassen:

### Sichtbarkeit von Geschlechterdiversität in der Forschung verbessern.

Die methodischen Herausforderungen rechtfertigen nicht per se einen Verzicht auf die Erhebung geschlechtlicher Diversität. Im Gegenteil erscheint es notwendig, diesen Gruppen in bevölkerungsweiten Erhebungen zur Sichtbarkeit zu verhelfen. Dies ist wichtig, um abzubilden, ob und wie viele inter*/intersexuelle und trans*/transsexuelle oder nichtbinäre Personen tatsächlich an Befragungen teilnehmen, und somit Daten zur Prävalenz der Geschlechterdiversität zu sammeln [26].

### Transparenz zum Umgang mit der Variablen „Geschlecht“ in Studien herstellen.

Wenn neben einer Darstellung der zahlenmäßigen Repräsentanz der einzelnen Gruppen eine auf sie bezogene Auswertung einzelner Variablen nicht möglich ist und sie in der Auswertung nicht berücksichtigt wird, geht der Informationsgehalt einzelner Datensätze vollständig verloren. Aus diesem Grund werden in einigen Studien Daten nach einem vorher festgelegten Schema randomisiert anderen Gruppen zugeordnet. Das hat den Vorteil, dass der Informationsgehalt der Datensätze, die nach anderen Ordnungskriterien auswertbar wären (z. B. bei Filterung nach Alter, Bildungsstatus), erhalten bleibt. Bei der Formulierung von Einverständniserklärungen müsste in diesem Fall möglicherweise darauf hingewiesen werden, dass nicht alle bzw. welche personenbezogenen Merkmale erhoben, aber im Falle von zu kleinen Stichprobengrößen nicht entsprechend der Erhebungskategorie ausgewertet werden können. Solch ein Vorgehen wurde allerdings als „analytic microaggressions“ bezeichnet [27]. Microagressions werden definiert als alltägliche, oft unbewusste verbale, nonverbale oder umgebungsbedingte Kränkungen und Beleidigungen [28], die auch im Kontext sexueller Minderheiten zu finden sind [29] und sich negativ auf deren mentale Gesundheit auswirken können [30]. Befragten die Möglichkeit zu geben, ihre Identität selbstbestimmt zum Ausdruck zu bringen, diese beim Generieren von Schlussfolgerungen allerdings auszuschließen, könnte als kränkend und verletzend empfunden werden. Unklar ist daher, inwiefern solch ein Vorgehen das nachfolgende Antwortverhalten der Befragten (negativ) beeinflussen würde. Unabhängig davon, ob Wissenschaftler:innen sich dazu entscheiden, Daten von inter*/intersexuellen, trans*/transsexuellen oder nichtbinären Befragten von bestimmten Analysen auszuschließen oder diese randomisiert umzuverteilen, sollte das Vorgehen immer sorgfältig beschrieben werden (vgl. Cameron und Stinson [16]). Das könnte Wissenschaftler:innen dabei helfen, ihre Vorgehensweisen gegenseitig einzusehen, zu bewerten, zu diskutieren und so idealerweise zu einer Best Practice zu gelangen. Ein Beispiel dafür ist die Initiative „AdvanceGender“ vom RKI, die sich der Fragestellung widmet, wie sich die Gesundheitsberichterstattung stärker geschlechtersensibel und intersektional ausrichten kann, und einen Austausch zu der Thematik zwischen Wissenschaftler:innen stimuliert [31]. Weitere Initiativen dieser Art sind wünschenswert.

### Studien mit Menschen diverser Geschlechteridentität etablieren.

Sollen Aussagen zu spezifischen Bevölkerungsgruppen generiert werden, ist eine bevölkerungsweite Repräsentativerhebung möglicherweise nicht der passende Zugang. Stattdessen könnten sich Untersuchungen in der jeweiligen Population empfehlen, um zielgruppenspezifische und auswertbare Daten zu generieren. Einige Autor:innen haben sich bereits für diesen Weg entschieden und Stichprobengrößen erhalten, die konkretere Rückschlüsse auf verschiedene Gruppen ermöglichen [32–34].

### Offene Antwortmöglichkeiten zur Selbstbeschreibung anbieten.

Basierend auf der Empfehlung der GenIUSS Group [25], wurde in der GeSiD-Studie Geschlecht anhand der beiden Fragen zum Geburtsgeschlecht und zur Geschlechtszugehörigkeit erhoben. Hierbei handelt es sich um einen zweistufigen Ansatz, der sich als der momentan vorherrschende Ansatz identifizieren lässt [35–37]. Die Verwendung einer offenen Antwortmöglichkeit in der zweiten Frage stimmt auch mit neueren Empfehlungen überein, wie zum Beispiel von Spiel und Kolleg:innen [38], die ähnliche Antwortkategorien sowie ebenfalls eine offene Antwortmöglichkeit („prefer to self-describe: [open answer]“) empfehlen. Dies hat den Vorteil, dass Personen, die sich mit den vorgegebenen Antwortkategorien nicht identifizieren können, ihre Identität durch das offene Antwortformat individuell ausdrücken und selbst definieren können. Wissenschaftler:innen, die sich für den Gebrauch einer solchen Frage entscheiden, müssen dann allerdings die offenen Antworten codieren und transparent machen, wie sie mit den Daten in Analysen umgehen. Die 4 Antworten, die die Befragten der GeSiD-Studie auf diese offene Frage gaben, sind vielfältig und eher schwierig zu kategorisieren. Eine Best-Practice-Empfehlung zum Umgang mit offenen Antworten dieser Art besteht zum jetzigen Zeitpunkt nach unserem Wissensstand nicht. Außerdem ist auch bei der ersten Frage nach dem Geburtsgeschlecht Vorsicht geboten – durch den in Deutschland mittlerweile ergänzten Geschlechtereintrag wären zum Jetztstand 5 Optionen abzufragen: „männlich“, „weiblich“, „divers“, „kein Eintrag“ und „keine Angabe“. Der je aktuelle Rechtsstand ist also einzubeziehen, erschwert dann jedoch historische oder internationale Vergleiche.

### Genderskalen weiterentwickeln.

Eine andere Möglichkeit wäre es, Geschlecht nicht als kategoriales Merkmal, sondern als eine kontinuierliche Variable in Form einer Skala zu erfassen [39]. Die meistgenutzte Genderskala ist das Bem Sex Role Inventory (BSRI; [40]), das nicht erfragt, *ob* die Befragten sich einer bestimmten (vorgegebenen) Antwortkategorie zuordnen können, sondern vielmehr *inwieweit* diese sich auf einem Kontinuum mit bestimmten femininen oder maskulinen Eigenschaften (z. B. „sachlich“, „feinfühlig“, „kraftvoll“) identifizieren. Basierend auf 60 Items, können Befragte auf Basis ihrer Antworten in 4 Gruppen kategorisiert werden: 1. maskulin (starke Selbstzuschreibung maskuliner, geringe Selbstzuschreibung femininer Eigenschaften), 2. feminin (starke Selbstzuschreibung femininer, geringe Selbstzuschreibung maskuliner Eigenschaften), 3. androgyn (starke Selbstzuschreibung sowohl maskuliner als auch femininer Eigenschaften) und 4. undifferenziert (geringe Selbstzuschreibung sowohl maskuliner als auch femininer Eigenschaften). Es hat sich gezeigt, dass Genderskalen sich besser dazu eignen, um zum Beispiel Einstellungen oder Verhaltensweisen von Personen vorherzusagen als kategoriale Antworten [41]. Der Nachteil von Genderskalen ist allerdings, dass diese sehr lang sind und oftmals historischem und kulturellem Wandel unterliegen bezüglich stereotyper Betrachtungen, was als typisch feminin oder als typisch maskulin angesehen wird [39]. Auch besteht die Gefahr, dass ein binäres Geschlechterkonzept hiermit indirekt fortgeschrieben wird. Hier gilt, dass die Wissenschaftler:innen in Abhängigkeit von der Fragestellung, dem Forschungsgegenstand und unter Berücksichtigung von (Zeit‑)Kosten und Nutzen entscheiden müssen, welche Art von Frage sich am besten für die jeweilige Befragung eignet.

### Partizipative Geschlechterforschung vorantreiben.

Obwohl es die oben erläuterten Möglichkeiten zur Geschlechtserhebung gibt, ist zu bemängeln, dass diese aus einem top-down (von oben nach unten) ähnlichen Ansatz resultierten: Wissenschaftler*innen formulieren Geschlechterfragen und lassen trans*, inter* und nichtbinäre Personen bewerten, inwieweit sie diese Formulierungen akzeptieren. Eine andere Vorgehensweise könnte es deshalb sein, insbesondere (jedoch nicht ausschließlich) trans*, inter* und nichtbinäre Personen offen zu fragen, wie diese gerne zu ihrem Geschlecht befragt werden wollen, ohne selbige durch vorgefertigte Fragen in eine bestimmte Richtung zu lenken. Dies würde eher einem Bottom-up-Ansatz ähneln. Studien, die sich dieser Fragestellung widmen, sind zum jetzigen Zeitpunkt noch rar. Eine Studie hat allerdings gezeigt, dass der weitverbreitete zweistufige Ansatz, der auch in der GeSiD-Studie verwendet wurde, von einigen Befragten selbst vorgeschlagen wird, von anderen Befragten allerdings als „schmerzhaft“ empfunden wird („It should ask [if] we are trans, rather than our birth assignment, because having to identify in any way with our sex assigned at birth can be very painful“ [42]). Ein Umdenken zu einem Bottom-up-Vorgehen sollte daher stimuliert werden, da dieses dazu verhelfen könnte, sowohl die Genauigkeit der Geschlechtserhebung zu verbessern als auch zielgruppengerechter vorzugehen.

## Fazit

Es lässt sich festhalten, dass das Bewusstsein für die Vielfalt der Geschlechter seinen Eingang in die empirische Forschung gefunden hat. Selbiges gilt für das Problem der Operationalisierung des Merkmals „Geschlecht“ in Befragungen. Dies ist ein erster notweniger Schritt in Richtung Veränderung. Anhand der GeSiD-Studie haben wir aufgezeigt, dass viele Fragen zur Operationalisierung und Repräsentation von Geschlecht noch nicht beantwortet werden können bzw. der Versuch der Beantwortung neue Herausforderungen aufwirft. Dies sollte Wissenschaftler:innen jedoch nicht davon abhalten, ihre Forschung inklusiv zu gestalten. Wissenschaft hat den Anspruch, Forschung von höchster Qualität durchzuführen, um Ungleichheiten anzugehen, insbesondere in Bezug auf diskriminierte Minderheiten. Dafür sind ein offener Austausch und Diskurs notwendig, den wir mit diesem Artikel anregen möchten.
